# The Effect of the Prognostic Nutritional Index and Triglyceride–Glucose Index for Predicting Mortality in Patients with Stroke

**DOI:** 10.3390/diagnostics16213612

**Published:** 2026-11-01

**Authors:** Muhammed Ekmekyapar, Tuba Ekmekyapar, Seda Tuğrul, Cem Doğan, Pelin Kaya, Zeynep Ceyhan

**Affiliations:** 1 Department of Emergency Medicine, Elazığ Fethi Sekin City Hospital, 23280 Elazığ, Türkiye; seda_tugrul@hotmail.com (S.T.); p.kayaa2323@gmail.com (P.K.); drzeynepcyh@gmail.com (Z.C.); 2 Department of Neurology, Faculty of Medicine, Malatya Turgut Özal University, 44210 Malatya, Türkiye; dr.tuba_polat@hotmail.com; 3 Department of Emergency Medicine, Malatya Education and Research Hospital, 44330 Malatya, Türkiye; cemdogan0062@gmail.com

**Keywords:** stroke, mortality, early mortality, PNI, TyG

## Abstract

**Background/Objectives:** Stroke is an acute condition in which impaired cerebral perfusion causes focal or global neurological dysfunction. Serious complications are a major contributor to morbidity and mortality in patients with stroke. Malnutrition is common in this population, and nutritional markers, including the prognostic nutritional index (PNI), may be useful for early risk assessment of stroke-associated infections. Insulin resistance (IR) is also an emerging risk factor for stroke and has been associated with poor prognosis through various metabolic and inflammatory pathways. The triglyceride–glucose (TyG) index has been proposed as a reliable surrogate marker of IR. Various biochemical and clinical parameters are used to assess prognosis in patients with stroke. We investigated the prognostic value of the TyG index and PNI for mortality and early mortality, defined as death within the first 30 days, in these patients. **Methods:** After application of the exclusion criteria, 415 patients with stroke were included. Demographic characteristics, including age and sex, and comorbidities were reviewed. Complete blood count, biochemical parameters, C-reactive protein, blood gas measurements, and lactate levels were recorded. Brain computed tomography and diffusion-weighted magnetic resonance imaging findings were reviewed to classify patients into ischemic and hemorrhagic stroke subgroups. Patients were grouped as survivors or nonsurvivors, and deaths within the first 30 days were classified as early mortality. PNI and TyG index values were calculated using the following formulas: PNI = 10 × serum albumin (g/dL) + 0.005 × total lymphocyte count (cells/mm^3^); TyG index = ln [fasting triglycerides (mg/dL) × fasting blood glucose (mg/dL)/2]. **Results:** Of the 415 patients included, 389 (93.7%) had ischemic stroke and 26 (6.3%) had hemorrhagic stroke. The median age was 74 years (IQR, 65.0–81.0), and the sex distribution was balanced: 207 patients (49.9%) were women and 208 (50.1%) were men. The PNI was lower in nonsurvivors than in survivors in the overall stroke cohort (ischemic and hemorrhagic stroke combined; 40.86 ± 8.02 vs. 46.88 ± 6.85, *p* < 0.001), the ischemic stroke subgroup (41.12 ± 8.77 vs. 46.82 ± 6.83, *p* = 0.001), and the hemorrhagic stroke subgroup (39.79 ± 3.79 vs. 48.17 ± 7.32, *p* = 0.006). The PNI was also lower in patients who died within the first 30 days than in survivors in the overall cohort (41.86 ± 8.42 vs. 46.88 ± 6.85, *p* < 0.001), the ischemic stroke subgroup (42.46 ± 8.90 vs. 46.82 ± 6.83, *p* = 0.002), and the hemorrhagic stroke subgroup (38.60 ± 4.35 vs. 48.17 ± 7.32, *p* = 0.012). **Conclusions:** Biomarkers calculated using simple hematological parameters have a significant impact on mortality and early mortality in stroke patients (both ischemic and hemorrhagic stroke). When we examined the effects of these TyG and PNI markers on mortality in stroke patients in our study, we observed that the PNI had a significant impact on mortality. Although our findings suggest that the PNI independently predicts mortality or could guide risk assessment in the emergency department, we consider this an exploratory observation, as stroke severity measures—such as the NIHSS—and relevant treatment data were not included in our analyses. However, we believe that single-point TyG values do not carry sufficient significance regarding mortality; therefore, future research should take into account cumulative, repeated TyG measurements obtained during patient follow-up.

## 1. Introduction

Stroke is an acute condition in which impaired cerebral perfusion causes focal or global neurological dysfunction. It is broadly classified as ischemic or hemorrhagic, with approximately 84% of strokes being ischemic. More than 13.7 million strokes occur worldwide each year, resulting in 5.5 million deaths annually. By causing death, dementia, and disability, stroke reduces quality of life and imposes substantial economic and social burdens [1]. Cerebrovascular disease remains a major contributor to the global burden of disease. Most survivors experience disability and neurological damage and are at increased risk of cognitive decline and systemic vascular disease. Patients with cerebrovascular disease admitted to an intensive care unit (ICU) have more severe impairment of consciousness, more complex clinical presentations, and higher mortality rates [2].

Cerebral small vessel disease (CSVD) is one of the primary causes of vascular cognitive impairment and stroke. CSVD generally progresses silently. Particularly in their early stages, white matter hyperintensities (WMHs) and enlarged perivascular spaces (ePVSs) are incidentally detected during neuroimaging in symptomatic adults. It is anticipated that the presence of these occult lesions increases the risk of stroke, depression, and long-term functional dependence. Furthermore, the current understanding of CSVD pathophysiology encompasses a range of microvascular dysfunctions—such as endothelial damage, blood–brain barrier (BBB) disruption, chronic hypoperfusion, and low-grade neuroinflammation—that develop over years before becoming radiologically or clinically apparent [3].

Assessing prognosis in patients with stroke is essential for optimizing treatment strategies and allocating resources efficiently. Accordingly, prognostic models incorporating various biochemical and clinical parameters have been developed. An increased inflammatory burden plays an important role in stroke pathophysiology. Inflammatory processes contribute to both injury and repair, underscoring the importance of inflammation in determining stroke outcomes. Other neurological diseases are also associated with varying degrees of inflammation [4]. Stroke carries high rates of morbidity and mortality, to which serious complications contribute substantially. Malnutrition is common in patients with stroke and is frequently overlooked in hospitalized patients. It worsens prognosis, increases mortality, and impairs immune function. The resulting reduction in resistance to pathogenic microorganisms can make infections more difficult to treat. These changes increase the risk of infection in patients with stroke, while the immunosuppressive effects of brain injury further facilitate infection. Stroke-associated infections are therefore both common and serious complications [5].

Nutritional status may be a reliable indicator of the risk of infection, and nutritional markers may be useful for early risk assessment of stroke-associated infections. The prognostic nutritional index (PNI) is a novel index of nutritional and inflammatory status calculated from the lymphocyte count and serum albumin level [5]. It reflects both nutritional status and overall health and has demonstrated prognostic value, particularly in chronic diseases and malignancies. Associations between the PNI and inflammatory conditions, including diabetic nephropathy and infections, have also been reported. The established relationship between nutritional indices and inflammation highlights the potential utility of the PNI in assessing inflammatory processes in patients with stroke [4].

Identifying patients at high risk is essential for predicting short-term outcomes and providing appropriate treatment. Readily available and safe markers are increasingly needed to improve prognostic assessment and risk stratification in stroke. Insulin resistance (IR) is an emerging risk factor for stroke and has been associated with poor prognosis through various metabolic and inflammatory pathways. The triglyceride–glucose (TyG) index, calculated from fasting plasma glucose and triglyceride levels, has been proposed as a reliable surrogate marker of IR. Compared with other markers that estimate IR directly or indirectly, the TyG index is more cost-effective and readily available in routine clinical practice [6]. Following initial studies of its use in diabetes, numerous publications have reported its utility in other conditions. The TyG index has been associated with arterial stiffness, cardiovascular disease (CVD), and the severity of metabolic syndrome, a cluster of metabolic abnormalities characterized by hypertension, dyslipidemia, obesity, and dysregulated glucose metabolism. Stroke is a leading cause of death and disability in low- and middle-income countries, and improving post-stroke outcomes is a global priority. The relationship of the TyG index with stroke risk and stroke subtypes has been investigated [1]. Patients with a higher TyG index have been reported to have a 1.30-fold higher risk of ischemic stroke [7]. The available literature provides strong support for the TyG index as a potential independent marker of recurrent stroke and clinical outcomes [1].

In studies concerning stroke, 30-day mortality has been evaluated [8,9], and 30-day mortality was considered early mortality [9]. Furthermore, in other studies, mortality occurring within the first 30 days has been defined as early mortality [10,11]. In this study, we investigated the prognostic value of the TyG index and PNI for mortality and early mortality, defined as death within the first 30 days, in patients with stroke, a condition frequently encountered in emergency departments.

## 2. Materials and Methods

### 2.1. Patient Selection

The study was initiated after approval had been obtained from the Non-Interventional Research Ethics Committee of Elazığ Fethi Sekin City Hospital on 7 November 2024 (session no. 2024/3-10). The study was conducted as a retrospective observational cohort study. A total of 510 patients older than 18 years who presented to the Department of Emergency Medicine at Elazığ Fethi Sekin City Hospital between 1 November 2023 and 31 October 2024 and were admitted to the neurology ward or neurological ICU with suspected stroke were identified.

Our study included patients with ischemic or hemorrhagic stroke who presented to the emergency department, were diagnosed with a stroke, and were subsequently hospitalized. These patients required hospitalization due to a stroke; they did not need to be hospitalized for any other comorbid condition. Patients with only ischemic stroke or only hemorrhagic stroke were included in the study. Patients were not included in the study consecutively. Patients with incomplete data (32 patients), patients with transient ischemic attack (23 patients), patients with subarachnoid hemorrhage (12 patients), patients with an intracranial space-occupying lesion (11 patients), patients with hemorrhage into a mass (9 patients), and patients with hemorrhagic transformation of an ischemic lesion (8 patients) were excluded from the study. After excluding 95 patients, the study was completed with a total of 415 patients, consisting of 389 patients with ischemic stroke and 26 patients with hemorrhagic stroke.

### 2.2. Data Collection and Study Design

Demographic characteristics, including age and sex, comorbidities, and history of stroke were reviewed. Comorbidities included hypertension (HT), coronary artery disease (CAD), atrial fibrillation (AF), diabetes mellitus (DM), chronic renal failure (CRF), chronic obstructive pulmonary disease (COPD), hyperlipidemia, and malignancy. Complete blood count parameters (white blood cell [WBC] count, hemoglobin, lymphocyte count, neutrophil count, and platelet count), biochemical parameters (glucose, urea, creatinine, albumin, total bilirubin, total cholesterol, triglycerides, high-density lipoprotein [HDL] cholesterol, and low-density lipoprotein [LDL] cholesterol), C-reactive protein (CRP), blood gas measurements, and lactate levels were recorded. Brain computed tomography and diffusion-weighted magnetic resonance images were reviewed, and patients were classified as having ischemic or hemorrhagic stroke on the basis of the imaging findings. The total number of days spent in the ward and ICU was recorded. Mortality status was reviewed, and patients were grouped as survivors or nonsurvivors. Deaths within the first 30 days were classified as early mortality.

The PNI value is derived from the blood test results of patients at the time of their presentation to the emergency department. However, since the TyG index must be calculated using fasting glucose and fasting triglyceride levels, the TyG values were determined based on the fasting glucose and fasting triglyceride measurements obtained at the earliest possible time following the patients’ hospitalization.

The PNI and TyG index values of the patients were calculated in accordance with the literature using the following formulas: PNI = 10 × serum albumin (g/dL) + 0.005 × total lymphocyte count (per mm^3^), and TyG = ln [fasting triglycerides (mg/dL) × fasting blood glucose (mg/dL)/2] [2,4,5,6,7]. In our hospital, albumin levels are measured in g/L. For the PNI calculation, albumin values were converted to g/dL. Lymphocyte counts were expressed in units of 10^3^/mm^3^. Fasting triglyceride and fasting glucose values were both expressed in mg/dL.

**Figure 1:**
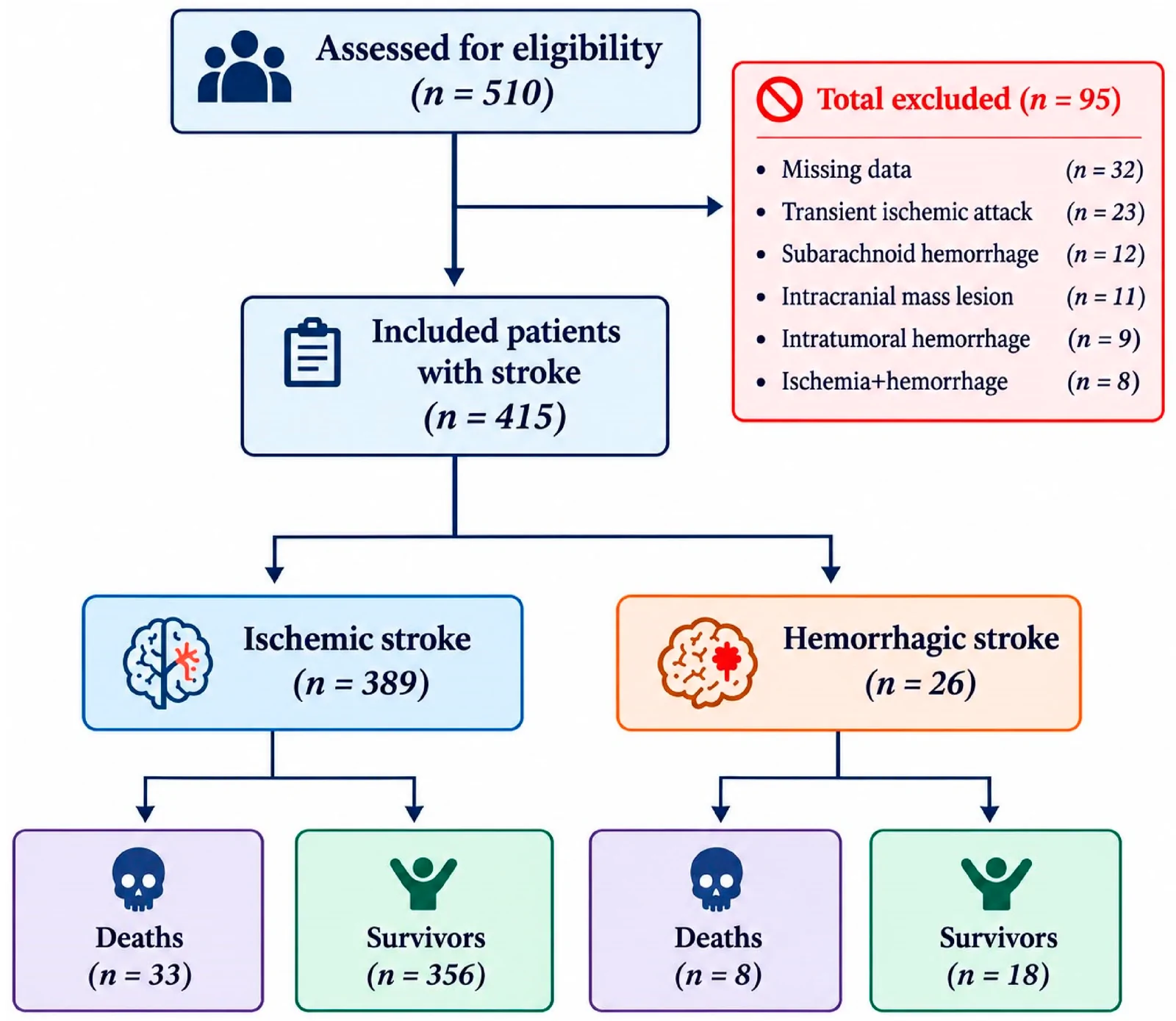
Patient selection and allocation flowchart.

The medical records of the patients throughout their hospitalization period were examined in detail. Death during a patient’s hospital stay was defined as mortality, while death within the first 30 days of hospital admission was defined as early mortality. Patients who did not die during their hospital stay and were subsequently discharged were classified as survivors. The total cohort consisted of 415 patients, who were grouped into those who survived and those who died. As for early mortality, analyses were conducted based on 406 patients. In this case, patients were categorized into those who survived and those who experienced mortality within the first 30 days (early mortality). Nine patients who died after 30 days were not included in this analysis. Stroke subtypes were divided into two categories: ischemic and hemorrhagic. Of a total of 415 stroke patients, 389 had ischemic strokes and 26 had hemorrhagic strokes. Of the 415 stroke patients, 374 survived, while 41 died. Of 389 ischemic stroke patients, 356 survived, while 33 died. Of the 26 patients with hemorrhagic stroke, 18 survived, while 8 died. Patient selection and allocation flowchart is shown in Figure 1.

### 2.3. Statistical Analysis

Statistical analyses were performed using the Statistical Package for the Social Sciences (SPSS) for Windows, version 22.0 (IBM SPSS Inc., Chicago, IL, USA). Normality was assessed using the Kolmogorov–Smirnov test. Normally distributed continuous variables are presented as mean ± standard deviation (SD), and nonnormally distributed variables as median (IQR). Categorical variables are presented as counts and percentages. Between-group comparisons of continuous variables were performed using the independent-samples Student’s *t* test for normally distributed data and the Mann–Whitney U test for nonnormally distributed data. Categorical variables were compared using the chi-square test or Fisher’s exact test. Comparisons were made between patients with and without in-hospital mortality and between the ischemic and hemorrhagic stroke groups. Because the PNI was significantly associated with mortality, receiver operating characteristic (ROC) analysis was performed to assess its prognostic performance. Sensitivity, specificity, positive and negative predictive values, and accuracy were calculated, and optimal cutoff values were identified. The area under the ROC curve (AUC) was assessed, and the optimal cutoff was determined using the Youden index. A *p* value < 0.05 was considered statistically significant.

To evaluate whether the PNI was associated with in-hospital mortality independently of other factors, univariable and multivariable binary logistic regression was performed. Because of the 41 deaths, the multivariable model was limited to four predictors (about 10 events per variable): PNI, age, stroke type, and C-reactive protein (CRP). Results are presented as odds ratios (OR) with 95% confidence intervals (CI). Model calibration was assessed with the Hosmer–Lemeshow test, explained variance with Nagelkerke R^2^, and discrimination with the area under the receiver operating characteristic (ROC) curve.

## 3. Results

### 3.1. Characteristics by Mortality Status

A total of 415 patients with ischemic or hemorrhagic stroke were included: 389 (93.7%) had ischemic stroke and 26 (6.3%) had hemorrhagic stroke. The median age was 74 years (IQR, 65.0–81.0), and the sex distribution was balanced, with 207 women (49.9%) and 208 men (50.1%). The most common comorbidities were HT (68.4%), DM (38.1%), CAD (33.7%), AF (22.2%), and hyperlipidemia (16.1%). Less common comorbidities included COPD (10.6%), CRF (3.6%), thyroid disease (3.6%), and malignancy (2.7%). Overall mortality was 9.9% (*n* = 41). Nonsurvivors were older than survivors, with median ages of 79 years (IQR, 71.0–87.5) and 73 years (IQR, 64.8–80.0), respectively (*p* = 0.001). The median length of hospital stay was also longer in nonsurvivors than in survivors: 12 days (IQR, 4.00–27.00) versus 6 days (IQR, 4.00–10.00; *p* = 0.004). Hemorrhagic stroke was more frequent among nonsurvivors (8 patients, 19.5%) than survivors (18 patients, 4.8%), whereas ischemic stroke was less frequent among nonsurvivors (33 patients, 80.5%) than survivors (356 patients, 95.2%; *p* = 0.002). HT was present in 70.6% of survivors (*n* = 264) and 48.8% of nonsurvivors (*n* = 20), representing a significant between-group difference (*p* = 0.005). DM was also more common in survivors (40.1%, *n* = 150) than in nonsurvivors (19.5%, *n* = 8; *p* = 0.010). CRF was present in 9.8% of nonsurvivors (*n* = 4) and 2.9% of survivors (*n* = 11), with the difference approaching statistical significance (*p* = 0.051). The groups did not differ in the prevalence of other comorbidities, including previous stroke, CAD, AF, COPD, hyperlipidemia, thyroid disease, and malignancy. The demographic and clinical characteristics of the overall stroke cohort, stratified by mortality status, are presented in Table 1.

Among patients with ischemic stroke (*n* = 389), mortality was 8.5% (*n* = 33). Nonsurvivors were older than survivors, with median ages of 79 years (IQR, 71.0–88.0) and 73 years (IQR, 65.0–80.8), respectively (*p* = 0.002). The median length of hospital stay was 9 days (IQR, 3.50–24.00) in nonsurvivors and 6 days (IQR, 4.00–9.00) in survivors (*p* = 0.033). HT and DM were more common in survivors, whereas CRF was more common in nonsurvivors. No differences were observed in the prevalence of other comorbidities, including previous stroke, CAD, AF, COPD, hyperlipidemia, thyroid disease, and malignancy. The demographic and clinical characteristics of patients with ischemic stroke, stratified by mortality status, are presented in Table 2.

Among patients with hemorrhagic stroke (*n* = 26), mortality was 30.8% (*n* = 8). Age, sex, and length of hospital stay were similar between survivors and nonsurvivors. A history of stroke was more common in nonsurvivors (50%, *n* = 4), whereas none of the survivors had a history of stroke (0%, n = 0; *p* = 0.005). No patients with hemorrhagic stroke had CRF or thyroid disease. No significant differences were identified in other demographic or clinical characteristics. The demographic and clinical characteristics of patients with hemorrhagic stroke, stratified by mortality status, are presented in Table 3.

### 3.2. Laboratory Findings by Mortality Status

In the overall cohort, the PNI was lower in nonsurvivors than in survivors (40.86 ± 8.02 vs. 46.88 ± 6.85, *p* < 0.001), whereas the TyG index was similar between groups. The median WBC count was 11.30 (IQR, 8.95–14.30) in nonsurvivors and 8.40 (IQR, 7.00–10.32) in survivors (*p* < 0.001). The neutrophil count was also significantly higher in nonsurvivors than in survivors: 8.77 (IQR, 6.12–11.89) versus 5.47 (IQR, 4.28–7.40; *p* < 0.001). Conversely, the lymphocyte count was lower in nonsurvivors, at 1.31 (IQR, 0.94–2.09), than in survivors, at 1.81 (IQR, 1.30–2.36; *p* = 0.002). Platelet counts differed significantly between nonsurvivors and survivors: 211 (IQR, 154–283) versus 241 (IQR, 202–294; *p* = 0.024). Median creatinine 0.97 mg/dL (IQR, 0.69–1.44) and urea 57 mg/dL (IQR, 38.5–90.0) levels were significantly higher in nonsurvivors (*p* = 0.003 and *p* < 0.01, respectively). Albumin levels were significantly lower in nonsurvivors, at 34 g/L (IQR, 29.5–37.5), than in survivors, at 38 g/L (IQR, 34.0–40.0; *p* < 0.001). CRP levels were 18.50 mg/L (IQR, 5.40–77.05) in nonsurvivors and 5.20 mg/L (IQR, 3.20–10.92) in survivors, a statistically significant difference (*p* < 0.001). Blood gas analysis showed higher lactate levels in nonsurvivors than in survivors: 1.60 mmol/L (IQR, 1.30–2.50) versus 1.40 mmol/L (IQR, 1.10–1.80; *p* = 0.011). Bicarbonate levels were lower in nonsurvivors than in survivors (25.4 [IQR, 21.4–28.3] vs. 26.1 [IQR, 24.0–28.5], *p* = 0.049). Other blood gas parameters (pH, pO_2_, and pCO_2_) were similar between groups. Laboratory values in the overall stroke cohort, stratified by mortality status, are compared in Table 4.

Among patients with ischemic stroke, the TyG index was similar between survivors and nonsurvivors, whereas the PNI was lower in nonsurvivors (41.12 ± 8.77 vs. 46.82 ± 6.83, *p* = 0.001). WBC and neutrophil counts and creatinine, urea, and CRP levels were higher in nonsurvivors, while lymphocyte counts and albumin levels were lower. Blood gas analysis showed lactate levels of 1.60 mmol/L (IQR, 1.25–2.70) in nonsurvivors and 1.40 mmol/L (IQR, 1.10–1.80) in survivors (*p* = 0.027). Bicarbonate levels were lower in nonsurvivors than in survivors (25.3 [IQR, 21.4–28.1] vs. 26.1 [IQR, 23.9–28.5], *p* = 0.039). Other blood gas parameters (pH, pO_2_, and pCO_2_) were similar between groups. Laboratory values in patients with ischemic stroke, stratified by mortality status, are compared in Table 5.

Among patients with hemorrhagic stroke, the TyG index was similar between survivors and nonsurvivors, whereas the PNI was lower in nonsurvivors (39.79 ± 3.79 vs. 48.17 ± 7.32, *p* = 0.006). Albumin levels were also lower in nonsurvivors. No statistically significant differences were observed in the other laboratory values, and blood gas parameters were similar between groups. Laboratory values in patients with hemorrhagic stroke, stratified by mortality status, are compared in Table 6.

### 3.3. Laboratory Findings by Early Mortality Status

The PNI was lower in patients in the early mortality group than in survivors (41.86 ± 8.42 vs. 46.88 ± 6.85, *p* < 0.001). WBC and neutrophil counts and urea, creatinine, and CRP levels were significantly higher in the early mortality group, whereas lymphocyte and platelet counts and albumin levels were lower. Blood gas analysis showed lower pH and bicarbonate levels in the early mortality group than in survivors: pH, 7.36 (IQR, 7.31–7.42) versus 7.39 (IQR, 7.35–7.42), *p* = 0.021; bicarbonate, 24.8 ± 4.3 versus 26.2 ± 3.6, *p* = 0.036. Lactate levels in the early mortality group were 1.90 mmol/L (IQR, 1.40–2.68), which was higher than in survivors (*p* < 0.01). Laboratory values in the overall stroke cohort, stratified by early mortality status, are compared in Table 7.

Laboratory parameters in patients with ischemic stroke were compared between those who died within the first 30 days (*n* = 27) and survivors (*n* = 356). The PNI was lower in the early mortality group (42.46 ± 8.90 vs. 46.82 ± 6.83, *p* = 0.002). WBC and neutrophil counts and urea, creatinine, and CRP levels were significantly higher in the early mortality group, whereas albumin levels and platelet counts were lower. Lactate levels were also higher in the early mortality group (1.90 [IQR, 1.40–2.70] vs. 1.40 [IQR, 1.10–1.80], *p* = 0.002). No significant differences were observed in the other blood gas parameters, including pH, pO_2_, pCO_2_, and HCO_3_. Laboratory values in patients with ischemic stroke, stratified by early mortality status, are compared in Table 8.

Among patients with hemorrhagic stroke, the TyG index was 8.94 ± 0.69 in survivors and 8.79 ± 0.74 in the early mortality group (*p* = 0.664). The corresponding PNI values were 48.17 ± 7.32 and 38.60 ± 4.35, respectively (*p* = 0.012). Other laboratory parameters were not compared because of the small sample size and the presence of only five patients in one group. Laboratory values in patients with hemorrhagic stroke, stratified by early mortality status, are compared in Table 9.

### 3.4. Performance of PNI for Predicting In-Hospital Mortality

**Figure 2:**
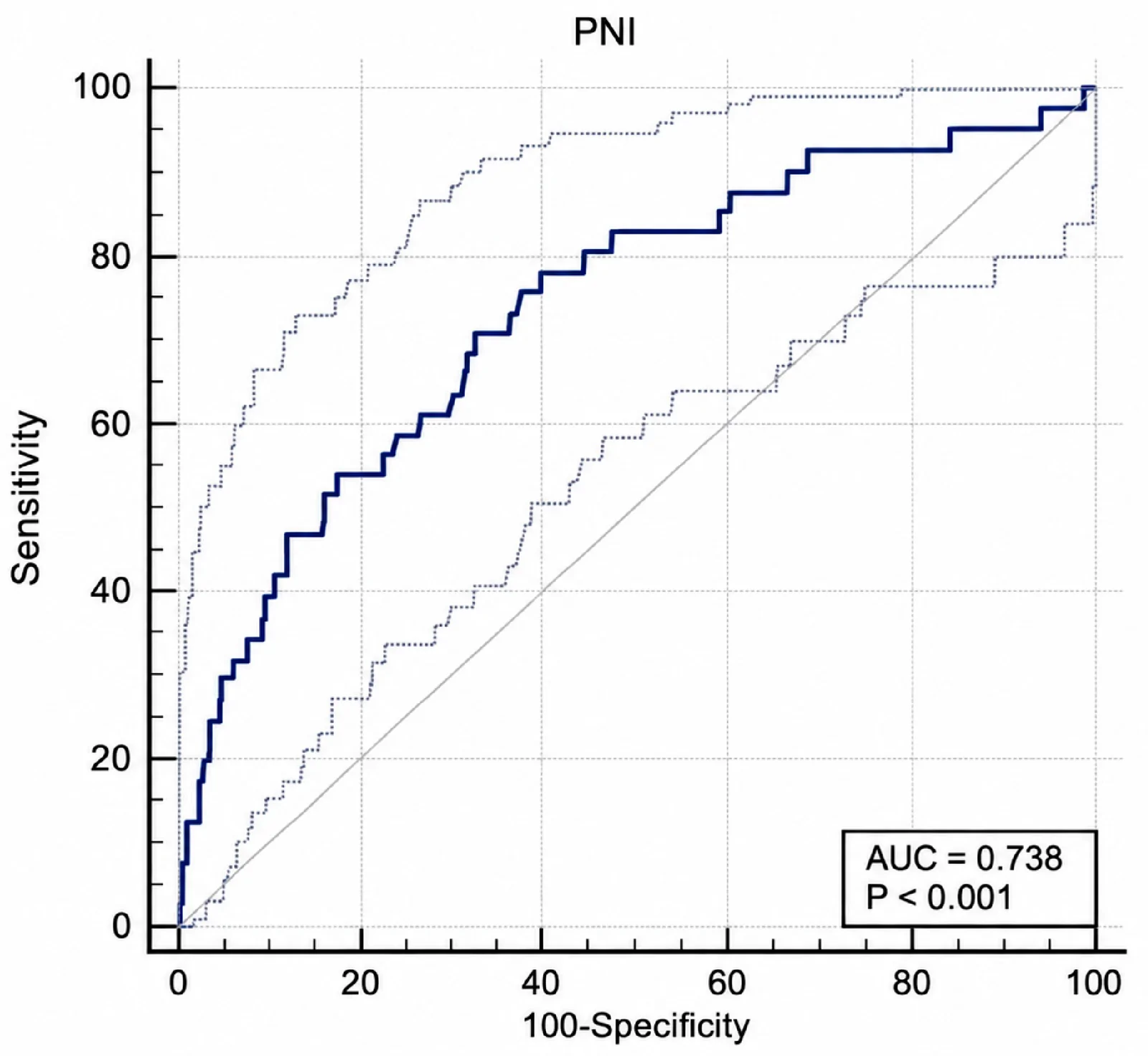
Receiver operating characteristic curve for PNI in predicting in-hospital mortality in the overall stroke cohort (ischemic and hemorrhagic stroke combined).

**Figure 3:**
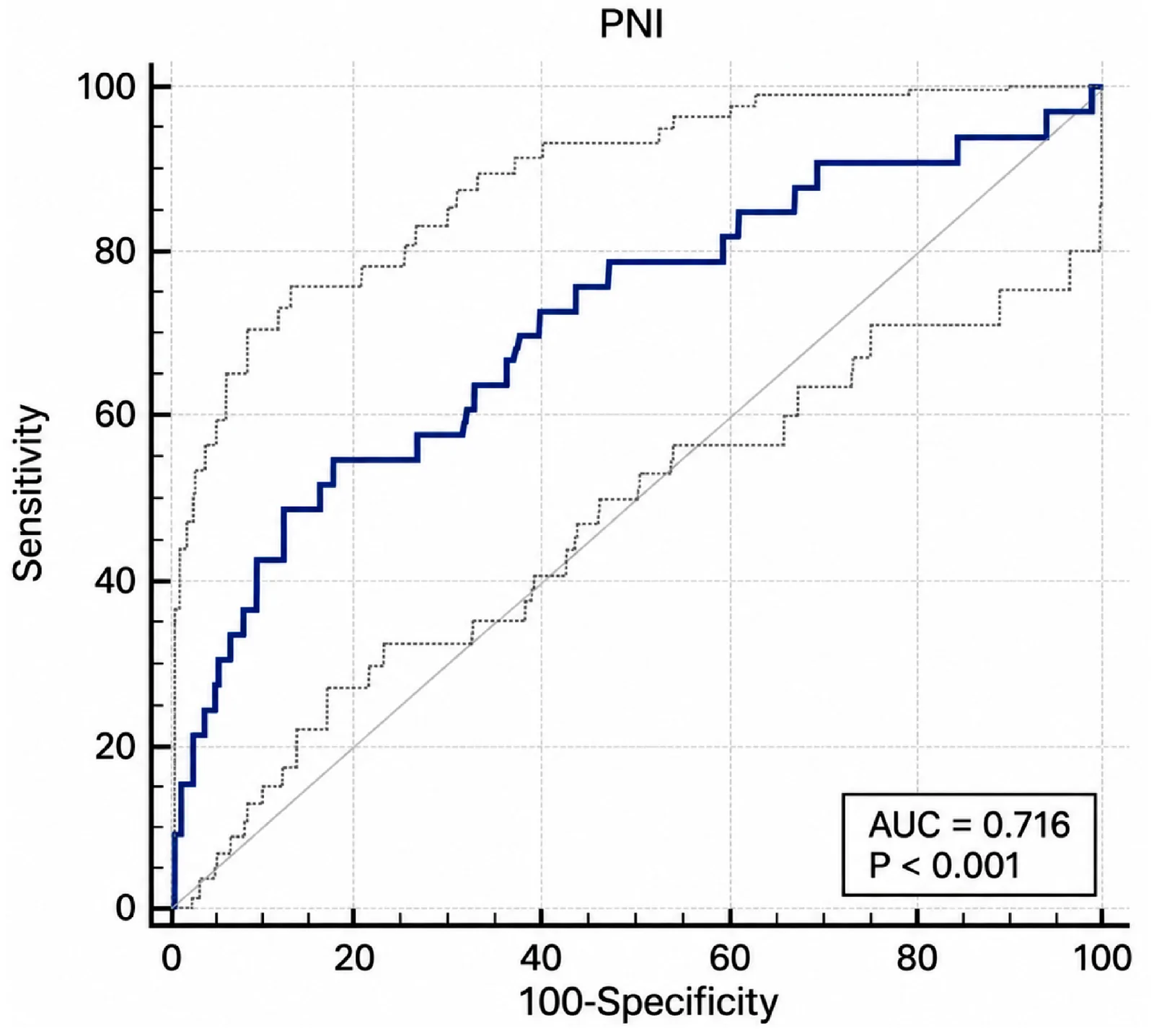
Receiver operating characteristic curve for PNI in predicting in-hospital mortality in patients with ischemic stroke.

**Figure 4:**
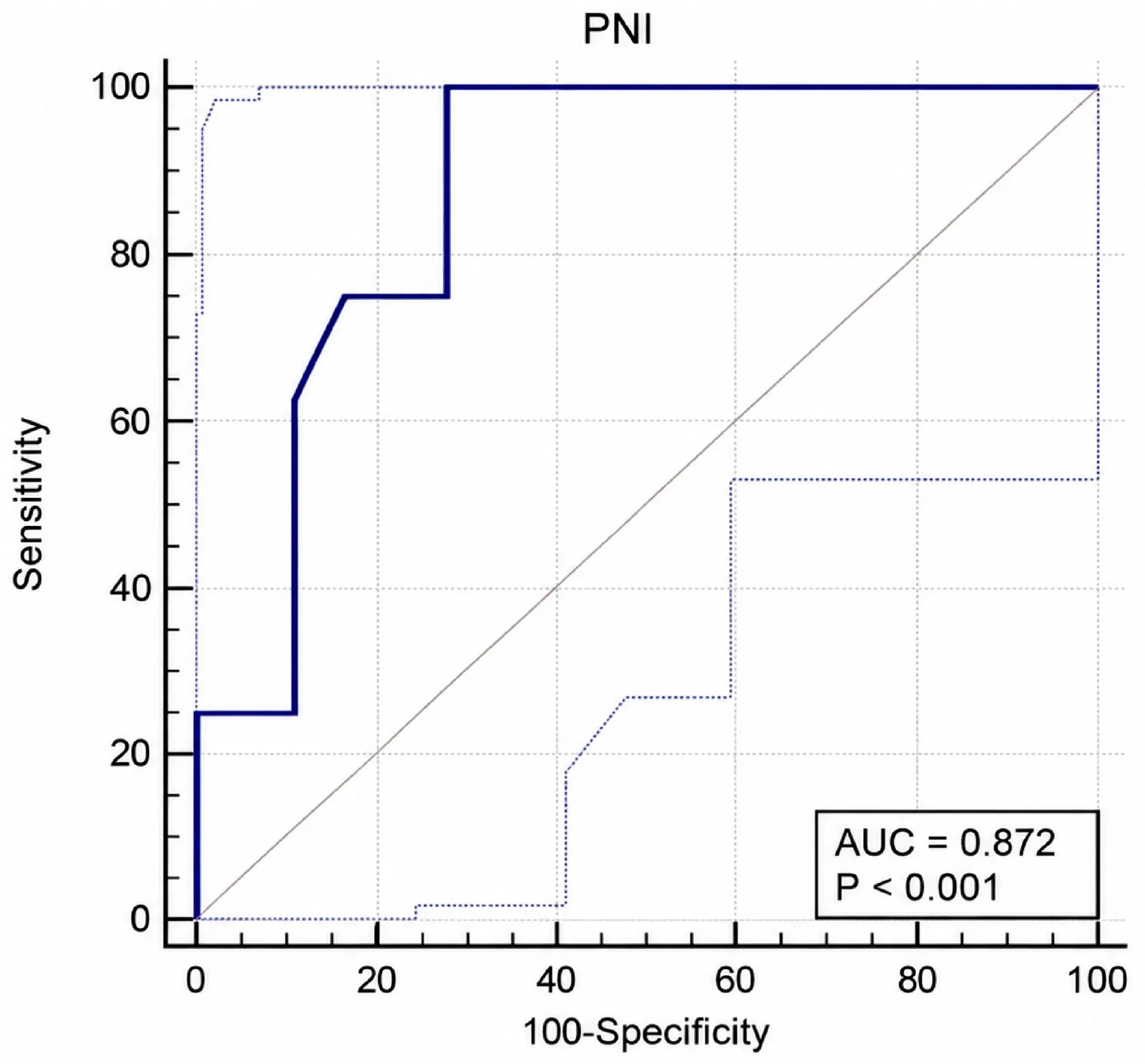
Receiver operating characteristic curve for PNI in predicting in-hospital mortality in patients with hemorrhagic stroke.

In the analysis of PNI performance for predicting in-hospital mortality, the AUC in the overall cohort was 0.738 (95% CI 0.652–0.824), with a cutoff of 45.3. At this threshold, sensitivity was 78%, specificity was 60.2%, the positive predictive value was 17.7%, and the negative predictive value was 96.2% (*p* < 0.001) (Figure 2). In the ischemic stroke subgroup, the AUC was 0.716 (95% CI 0.613–0.819), and the optimal cutoff was 40.5. At this threshold, sensitivity was 54.5%, specificity was 82.3%, the positive predictive value was 22.2%, and the negative predictive value was 95.1% (*p* < 0.001) (Figure 3). The PNI showed better discrimination for mortality in patients with hemorrhagic stroke, with an AUC of 0.872 (95% CI 0.735–1). The cutoff for this subgroup was 43.75, with 100% sensitivity, 72.2% specificity, a positive predictive value of 61.5%, and a negative predictive value of 100% (*p* < 0.001) (Figure 4). These analyses are presented in Table 10.

### 3.5. Univariable and Multivariable Logistic Regression Analysis of Factors Associated with In-Hospital Mortality

To determine whether the association between the PNI and in-hospital mortality was independent of other risk factors, we performed univariable and multivariable binary logistic regression (Table 11). The multivariable model included the PNI, age, stroke type, and C-reactive protein (CRP) and was limited to four predictors because of the 41 in-hospital deaths.

In univariable analysis, a lower PNI (OR 0.876 per 1-unit increase, 95% CI 0.831–0.923, *p* < 0.001), older age (OR 1.055 per year, 95% CI 1.021–1.089, *p* = 0.001), hemorrhagic stroke (OR 4.768, 95% CI 1.927–11.797, *p* < 0.001), and higher CRP (OR 1.013 per mg/L, 95% CI 1.007–1.019, *p* < 0.001) were associated with in-hospital mortality. In the multivariable model, all four variables remained significantly associated with mortality: PNI (adjusted OR 0.933, 95% CI 0.877–0.993, = 0.029), age (adjusted OR 1.054, 95% CI 1.014–1.095, *p* = 0.008), hemorrhagic stroke (adjusted OR 7.154, 95% CI 2.594–19.729, *p* < 0.001), and CRP (adjusted OR 1.010, 95% CI 1.004–1.016, *p* = 0.002). Each 1-unit increase in the PNI was associated with a 6.7% reduction in the odds of in-hospital mortality (adjusted OR 0.933, 95% CI 0.877–0.993), after adjustment for age, stroke type, and CRP (Table 11).

## 4. Discussion

Stroke is a leading cause of long-term disability in adults and the second leading cause of death worldwide [12]. The pathophysiological mechanisms underlying stroke are either ischemic or hemorrhagic. Ischemic stroke results from occlusion of an artery supplying the central nervous system, causing a rapid reduction in oxygen and glucose delivery to the affected brain region. Hemorrhagic stroke results from rupture of intracranial blood vessels and manifests as intracerebral or subarachnoid hemorrhage [13]. The increasing occurrence of stroke at younger ages also warrants attention [14].

CSVD is one of the leading causes of vascular cognitive impairment. The earliest stages of the disease remain clinically silent and are difficult to detect. Although WMHs are widely used neuroimaging markers, traditional cardiovascular risk scores and cognitive tests are insufficient in terms of their sensitivity to subclinical cerebrovascular damage [15]. ePVSs are increasingly recognized as early markers of CSVD. However, the underlying pathophysiology—specifically the role of perivascular fluid dynamics and circulating vascular biomarkers—is not yet fully understood. It is suggested that platelet-driven microvascular activation may be linked to early-stage perivascular dysfunction through alterations in perivascular fluid dynamics, and that this process occurs prior to the onset of significant white matter damage or cognitive impairment [16].

IR plays an important role in the pathogenesis of ischemic stroke by promoting atherosclerotic changes. It may contribute to ischemic stroke by increasing platelet adhesion, activation, and aggregation and by inducing hemodynamic disturbances [17]. IR is a metabolic state in which insulin-sensitive tissues become less responsive to insulin, resulting in disturbances of carbohydrate, lipid, and protein metabolism. It develops under the influence of several risk factors, including aging, alcohol consumption, smoking, a high-calorie diet, a sedentary lifestyle, and obesity. The contribution of IR to the development of conditions such as cardiovascular and cerebrovascular disease is now well established. IR also plays an important role in the pathogenesis and clinical outcomes of metabolic syndrome and type 2 DM [18]. It is a hallmark of metabolic syndrome and is recognized as a major risk factor for stroke. The TyG index, calculated as ln [fasting triglycerides (mg/dL) × fasting glucose (mg/dL)/2], has been proposed as a reliable surrogate marker of IR [19].

A study comprising 19 cohort studies and 10 case–control or cross-sectional studies, with a total of 11,944,688 participants, found that a higher TyG index was associated with an increased risk of cerebrovascular disease compared with a lower index. Each 1 mg/dL increase in the TyG index was associated with a 1.19-fold increase in cerebrovascular disease risk [20]. Another study assessed the differing associations of the TyG and TyG–body mass index (TyG-BMI) indices with short- and long-term mortality in critically ill patients with ischemic stroke. Of 1334 patients, 411 (30.81%) died within 1 year and 923 (69.19%) survived. The TyG index performed better in predicting short-term mortality at 30 and 90 days, whereas TyG-BMI performed better in predicting long-term mortality at 180 days and 1 year. Both indices were identified as valuable mortality predictors in critically ill patients with ischemic stroke. Mean age, WBC count, and creatinine level were significantly higher in nonsurvivors than in survivors (all *p* < 0.001): age, 78.31 versus 68.57 years; WBC count, 11.47 versus 10.12; and creatinine, 1.27 versus 1.04. Hemoglobin levels were significantly lower in nonsurvivors (11.60 vs. 12.54, *p* < 0.001). Triglyceride levels were higher in nonsurvivors (149.37 vs. 124.64), but the difference was not statistically significant. Glucose levels were significantly higher in nonsurvivors (154.94 vs. 129.79, *p* < 0.001) [21]. In our study, age, WBC count, and creatinine levels were significantly higher among nonsurvivors with ischemic stroke. Hemoglobin and triglyceride levels were lower and glucose levels were higher in nonsurvivors, although these differences were not statistically significant. Among patients with hemorrhagic stroke, nonsurvivors had higher age, WBC counts, and creatinine levels and lower hemoglobin, triglyceride, and glucose levels; none of these differences were significant.

A study investigating the relationship between TyG-BMI and 28-day mortality in 1362 critically ill patients with ischemic stroke reported a mean age of 68.41 ± 14.16 years; 47.50% of patients were men. Patients in the high TyG-BMI group had significantly higher 28-day in-hospital and ICU mortality rates [22]. A multicenter study of the associations between the TyG index, poor functional outcome at discharge, and in-hospital mortality included 3216 patients with ischemic stroke (1853 men and 1363 women). Their mean age was 68.6 ± 12.9 years, and the median TyG index was 8.65 mg/dL. During hospitalization, 105 patients (3.3%) died from various causes. A higher TyG index was associated with a higher risk of in-hospital death [6]. In our study, the median age of patients with ischemic stroke was 74 years (IQR, 66.0–81.0), and 197 (50.6%) were men. The TyG index was lower in nonsurvivors in both the ischemic and hemorrhagic stroke subgroups, but neither difference was statistically significant. We believe that regional differences in dietary habits may account for this finding.

A study of 733 critically ill patients with ischemic stroke reported a median age of 69 years (interquartile range [IQR], 58–79); 409 patients (55.4%) were men. The median TyG index was 8.90 (IQR, 8.53–9.34). In-hospital and ICU mortality rates were 19.0% and 14.9%, respectively. A higher TyG index was significantly associated with all-cause mortality, including both in-hospital and ICU mortality [23]. A population-based cohort study investigated the associations of baseline and long-term TyG indices with subsequent stroke and its subtypes in 97,653 participants with no previous history of stroke. Stroke occurred in 5122 participants, including 4277 cases of ischemic stroke, 880 cases of intracerebral hemorrhage, and 144 cases of subarachnoid hemorrhage. A linear association was observed between the baseline TyG index and stroke, with similar findings for ischemic stroke. However, no significant association was found between the baseline TyG index and the risk of intracranial hemorrhage. Over 11 years of follow-up, higher baseline and time-updated long-term cumulative mean TyG indices independently predicted stroke and ischemic stroke but not intracerebral hemorrhage [19]. In our study, the TyG index was calculated using fasting glucose and fasting triglyceride values obtained from patients at the earliest possible time point following hospitalization, and mortality was compared using this single measurement. The TyG index was not associated with mortality in the overall cohort or in either stroke subtype. Its lack of predictive value for mortality in patients with hemorrhagic stroke in our study is consistent with the literature.

Wang et al. identified the use of a single TyG index measurement, without analysis of changes over time or their long-term effects, as a limitation of previous studies. In a large prospective cohort study designed to characterize the relationship between changes in the TyG index and the subsequent risk of CVD and its subtypes, changes in the TyG index were identified as an independent predictor of CAD and its subtypes. An increase in the TyG index over time was associated with an increased risk of CAD, stroke, and myocardial infarction (MI). These findings emphasize the importance of monitoring temporal changes in the TyG index to identify individuals at high risk of developing CAD [24].

Most previous studies of the relationship between the TyG index and ischemic stroke have relied on a single measurement. However, the TyG index is influenced by numerous biological and pathological factors, including age, diet, and medication use. The relationship between the cumulative TyG index and incident ischemic stroke therefore remains incompletely defined. Wang et al. [7] evaluated the relationship between ischemic stroke and the cumulative TyG index, which incorporates both the index value and the duration of exposure to an elevated index. Age, sex, BMI, systolic and diastolic blood pressure, HDL cholesterol, LDL cholesterol, high-sensitivity CRP, smoking, physical exercise, lipid-lowering medication use, HT, and diabetes were significantly associated with ischemic stroke. The cumulative TyG index was also an independent risk factor for stroke. In analyses restricted to individuals without diabetes and those not taking antihypertensive, glucose-lowering, or lipid-lowering medications, longer exposure to an elevated TyG index was associated with increased stroke risk. Persistent exposure to an elevated TyG index over 6 years increased the risk of ischemic stroke by 38%. Longer exposure to a high cumulative TyG index was significantly associated with increased stroke risk, particularly ischemic stroke [7]. In our study, reliance on a single TyG index measurement during the acute phase may have limited its ability to predict mortality. The association between a high cumulative TyG index and increased ischemic stroke risk suggests that monitoring and controlling cumulative TyG exposure may be beneficial for ischemic stroke prevention.

The PNI is an objective nutritional index derived from serum albumin concentration and total lymphocyte count. These commonly measured, inexpensive biomarkers help assess nutritional, immune, and inflammatory status [25]. Serum visceral proteins, such as albumin and prealbumin, have traditionally been used as markers of nutritional status [26]. Low albumin, a serum visceral protein used to indicate malnutrition, may result from inadequate protein intake or reduced synthesis associated with increased inflammatory cytokine levels and liver failure. Hypoalbuminemia may also arise from renal losses, as in nephrotic syndrome, or gastrointestinal losses, as in protein-losing enteropathy [26,27,28]. Stenvinkel et al. reported that serum albumin levels below 3.5 g/dL were associated with a fourfold higher risk of death than higher levels and that levels below 3 g/dL indicated a critical condition [29]. Although the PNI was originally developed to assess risk in gastrointestinal surgery, it is now widely used as an indicator of survival and complications in cancer, cardiac disease, kidney disease, and severe infections [30]. In addition to its use in cerebrovascular disease, the PNI has been investigated in peripheral nerve disorders in relation to disease severity and long-term outcomes [31,32,33].

Several studies have examined the PNI in ischemic stroke. One study included 176 patients diagnosed with ischemic stroke in the emergency department, of whom 12 died and 164 survived. The PNI was significantly lower in nonsurvivors than in survivors (36.7 [28.7–40.5] vs. 46.05 [39.1–51.3], *p* < 0.001). Albumin levels were also significantly lower in nonsurvivors (3.31 [2.67–3.4] vs. 3.74 [3.39–4.21], *p* < 0.001) [4]. Another study of 158 patients with acute ischemic stroke reported an in-hospital mortality rate of 18%. Patients with a PNI > 380 were classified as not malnourished, whereas those with a PNI ≤ 380 were classified as malnourished. Patients with lower PNI scores were older and had significantly higher National Institutes of Health Stroke Scale (NIHSS) scores, as well as higher rates of AF, infection, mortality, and hospital admission [5]. In our study, significantly lower PNI and albumin values in the mortality and early mortality groups, both in the overall cohort and within the ischemic and hemorrhagic stroke subgroups, support their association with poor prognosis. Our findings are consistent with the published literature.

Xiang et al. reported that the PNI was independently associated with poor recovery at 3 months in patients with acute ischemic stroke (AIS) treated with intravenous thrombolysis (IVT; odds ratio, 2.250; confidence interval, 1.192–4.249; *p* = 0.012) [34]. In our study, a highly significant association between a lower PNI and mortality was also observed in the overall cohort and in both stroke subtypes. Although the PNI was higher among nonsurvivors with ischemic stroke than among those with hemorrhagic stroke, values in both groups were lower than the mean for the overall study population. The PNI was also significantly lower in patients who died within the first 30 days than in survivors (41.86 ± 8.42 vs. 46.88 ± 6.85, respectively; *p* < 0.001).

A study of 260 young patients with ischemic stroke, aged 18–45 years, classified patients according to favorable or unfavorable outcomes at 90 days. Outcomes were favorable in 144 patients and unfavorable in 116. The PNI was significantly lower in patients with unfavorable outcomes (41.04 ± 8.01 vs. 48.82 ± 8.19, *p* < 0.001). A history of diabetes or HT was also significantly more common in patients with unfavorable outcomes [35]. Consistent with these studies [4,5,34,35], our findings indicate that the PNI is a relevant marker of mortality and early mortality in stroke and its subtypes and has significant prognostic value in patients with stroke.

The single-center retrospective design of our study, the limited sample size of hemorrhagic stroke cases, and the fact that biomarker measurements during hospitalization were based on a single assessment are among our primary limitations. Furthermore, the failure to include validated clinical scales—such as the National Institutes of Health Stroke Scale (NIHSS), the Glasgow Coma Scale (GCS), or the modified Rankin Scale (mRS)—as well as the lack of documentation regarding infarct volume, hemorrhage volume, and the location of lesions, represent additional limitations of our study.

## 5. Conclusions

Patients with stroke generally first present to emergency departments, where diagnosis must be established promptly. In particular, patients with ischemic stroke who require time-sensitive intravenous thrombolysis or endovascular treatment must receive these interventions as quickly as possible and be managed by a multidisciplinary team. Nevertheless, mortality can occur despite treatment, making the assessment of prognosis and mortality risk essential. Numerous biomarkers have been investigated as predictors of mortality. In this study, we examined the prognostic value of the TyG index and PNI for mortality in patients with stroke. The TyG index did not predict mortality in our cohort. However, further studies should assess cumulative TyG indices derived from repeated measurements during hospitalization rather than relying on a single value. Unlike TyG, the PNI was a useful biomarker for predicting mortality in the overall stroke cohort and in the ischemic and hemorrhagic stroke subgroups. Our findings indicate that the PNI, which can be calculated from readily available blood test parameters, should be considered when assessing prognosis and mortality risk in patients with stroke, particularly at presentation to the emergency department. However, although our study indicates that the PNI independently predicts mortality or that it should guide risk assessment in the emergency department, we consider these findings to be exploratory, as stroke severity measures (such as the NIHSS) and relevant treatment data were not included in our analyses. In short, the PNI is associated with mortality; however, its independent and clinical predictive value requires further evaluation. Furthermore, we believe that for both biomarkers, it is necessary to conduct prospective, multicenter studies that involve larger patient cohorts—thereby avoiding limitations such as small sample sizes and single-point measurements—and that incorporate repeated measurements taken at multiple time points. Finally, in future studies, combining imaging markers indicative of chronic cerebrovascular damage with circulating prognostic biomarkers will be of potential value.

## Figures and Tables

**Table 1:** Demographic and clinical characteristics of the overall stroke cohort (ischemic and hemorrhagic stroke combined) by mortality status.

Characteristic	Total (*n* = 415)	Survivors (*n* = 374)	Nonsurvivors (*n* = 41)	*p*
Sex Female, n (%) Male, n (%)	207 (49.9) 208 (50.1)	184 (49.2) 190 (50.8)	23 (56.1) 18 (43.9)	0.401
Age (years), median (IQR)	74.0 (65.0–81.0)	73.0 (64.8–80.0)	79.0 (71.0–87.5)	0.001
Length of stay (days), median (IQR)	6.00 (4.00–11.00)	6.00 (4.00–10.00)	12.00 (4.00–27.00)	0.004
Stroke subtype Ischemic, n (%) Hemorrhagic, n (%)	389 (93.7) 26 (6.3)	356 (95.2) 18 (4.8)	33 (80.5) 8 (19.5)	0.002
History of stroke No, n (%) Yes, n (%)	327 (78.8) 88 (21.2)	296 (79.1) 78 (20.9)	31 (75.6) 10 (24.4)	0.599
HT No, n (%) Yes, n (%)	131 (31.6) 284 (68.4)	110 (29.4) 264 (70.6)	21 (51.2) 20 (48.8)	0.005
CAD No, n (%) Yes, n (%)	275 (66.3) 140 (33.7)	251 (67.1) 123 (32.9)	24 (58.5) 17 (41.5)	0.260
AF No, n (%) Yes, n (%)	323 (77.8) 92 (22.2)	291 (77.8) 83 (22.2)	32 (78) 9 (22)	0.965
DM No, n (%) Yes, n (%)	257 (61.9) 158 (38.1)	224 (59.9) 150 (40.1)	33 (80.5) 8 (19.5)	0.010
CRF No, n (%) Yes, n (%)	400 (96.4) 15 (3.6)	363 (97.1) 11 (2.9)	37 (90.2) 4 (9.8)	0.051
COPD No, n (%) Yes, n (%)	371 (89.4) 44 (10.6)	337 (90.1) 37 (9.9)	34 (82.9) 7 (17.1)	0.178
Hyperlipidemia No, n (%) Yes, n (%)	348 (83.9) 67 (16.1)	313 (83.7) 61 (16.3)	35 (85.4) 6 (14.6)	0.777
Thyroid disease No, n (%) Yes, n (%)	400 (96.4) 15 (3.6)	360 (96.3) 14 (3.7)	40 (97.6) 1 (2.4)	0.669
Malignancy No, n (%) Yes, n (%)	404 (97.3) 11 (2.7)	366 (97.9) 8 (2.1)	38 (92.7) 3 (7.3)	0.084

HT, hypertension; CAD, coronary artery disease; AF, atrial fibrillation; DM, diabetes mellitus; CRF, chronic renal failure; COPD, chronic obstructive pulmonary disease. Median (25th–75th percentile) compared with the Mann–Whitney U test; mean ± SD rows compare with the *t*-test.

**Table 2:** Demographic and clinical characteristics of patients with ischemic stroke by mortality status.

Characteristic	Total (*n* = 389)	Survivors (*n* = 356)	Nonsurvivors (*n* = 33)	*p*
Sex Female, n (%) Male, n (%)	192 (49.4) 197 (50.6)	174 (48.9) 182 (51.1)	18 (54.5) 15 (45.5)	0.533
Age (years), median (IQR)	74.0 (66.0–81.0)	73.0 (65.0–80.8)	79.0 (71.0–88.0)	0.002
Length of stay (days), median (IQR)	6.00 (4.00–10.00)	6.00 (4.00–9.00)	9.00 (3.50–24.00)	0.033
History of stroke No, n (%) Yes, n (%)	305 (78.4) 84 (21.6)	278 (78.1) 78 (21.9)	27 (81.8) 6 (18.2)	0.619
HT No, n (%) Yes, n (%)	120 (30.8) 269 (69.2)	103 (28.9) 253 (71.1)	17 (51.5) 16 (48.5)	0.007
CAD No, n (%) Yes, n (%)	253 (65) 136 (35)	235 (66) 121 (34)	18 (54.5) 15 (45.5)	0.186
AF No, n (%) Yes, n (%)	301 (77.4) 88 (22.6)	274 (77) 82 (23)	27 (81.8) 6 (18.2)	0.524
DM No, n (%) Yes, n (%)	237 (60.9) 152 (39.1)	211 (59.3) 145 (40.7)	26 (78.8) 7 (21.2)	0.028
CRF No, n (%) Yes, n (%)	374 (96.1) 15 (3.9)	345 (96.9) 11 (3.1)	29 (87.9) 4 (12.1)	0.010
COPD No, n (%) Yes, n (%)	347 (89.2) 42 (10.8)	320 (89.9) 36 (10.1)	27 (81.8) 6 (18.2)	0.149
Hyperlipidemia No, n (%) Yes, n (%)	325 (83.5) 64 (16.5)	297 (83.4) 59 (16.6)	28 (84.8) 5 (15.2)	0.833
Thyroid disease No, n (%) Yes, n (%)	374 (96.1) 15 (3.9)	342 (96.1) 14 (3.9)	32 (97) 1 (3)	0.797
Malignancy No, n (%) Yes, n (%)	379 (97.4) 10 (2.6)	348 (97.8) 8 (2.2)	31 (93.9) 2 (6.1)	0.205

**Table 3:** Demographic and clinical characteristics of patients with hemorrhagic stroke by mortality status.

Characteristic	Total (*n* = 26)	Survivors (*n* = 18)	Nonsurvivors (*n* = 8)	*p*
Sex Female, n (%) Male, n (%)	15 (57.7) 11 (42.3)	10 (55.6) 8 (44.4)	5 (62.5) 3 (37.5)	0.533
Age (years), mean ± SD	70.2 ± 11.6	68.2 ± 10.3	74.5 ± 13.8	0.209
Length of stay (days), median (IQR)	15.50 (5.75–24.50)	16.50 (5.00–22.50)	15.00 (9.00–38.75)	0.656
History of stroke No, n (%) Yes, n (%)	22 (84.6) 4 (15.4)	18 (100) 0 (0)	4 (50) 4 (50)	0.005
HT No, n (%) Yes, n (%)	11 (42.3) 15 (57.7)	7 (38.9) 11 (61.1)	4 (50) 4 (50)	0.683
CAD No, n (%) Yes, n (%)	22 (84.6) 4 (15.4)	16 (88.9) 2 (11.1)	6 (75) 2 (25)	0.563
AF No, n (%) Yes, n (%)	22 (84.6) 4 (15.4)	17 (94.4) 1 (5.6)	5 (62.5) 3 (37.5)	0.072
DM No, n (%) Yes, n (%)	20 (76.9) 6 (23.1)	13 (72.2) 5 (27.8)	7 (87.5) 1 (12.5)	0.628
CRF No, n (%) Yes, n (%)	26 (100) 0 (0)	18 (100) 0 (0)	8 (100) 0 (0)	
COPD No, n (%) Yes, n (%)	24 (92.3) 2 (7.7)	17 (94.4) 1 (5.6)	7 (87.5) 1 (12.5)	0.529
Hyperlipidemia No, n (%) Yes, n (%)	23 (88.5) 3 (11.5)	16 (88.9) 2 (11.1)	7 (87.5) 1 (12.5)	0.919
Thyroid disease No, n (%) Yes, n (%)	26 (100) 0 (0)	18 (100) 0 (0)	8 (100) 0 (0)	
Malignancy No, n (%) Yes, n (%)	25 (96.2) 1 (3.8)	18 (100) 0 (0)	7 (87.5) 1 (12.5)	0.308

**Table 4:** Comparison of laboratory values in the overall stroke cohort (ischemic and hemorrhagic stroke combined) by mortality status.

Characteristic	Total (*n* = 415)	Survivors (*n* = 374)	Nonsurvivors (*n* = 41)	*p*
TyG index, mean ± SD	9.12 ± 0.69	9.14 ± 0.70	8.95 ± 0.63	0.112
PNI, mean ± SD	46.29 ± 7.19	46.88 ± 6.85	40.86 ± 8.02	<0.001
WBC (×10^3^/mm^3^), median (IQR)	8.60 (7.10–10.60)	8.40 (7.00–10.32)	11.30 (8.95–14.30)	<0.001
Hemoglobin (g/dL), median (IQR)	13.90 (12.40–15.00)	13.90 (12.50–15.03)	12.70 (11.60–14.65)	0.049
Lymphocytes (×10^3^/mm^3^), median (IQR)	1.77 (1.25–2.35)	1.81 (1.30–2.36)	1.31 (0.94–2.09)	0.002
Neutrophils (×10^3^/mm^3^), median (IQR)	5.67 (4.39–7.80)	5.47 (4.28–7.40)	8.77 (6.12–11.89)	<0.001
Platelets (×10^3^/mm^3^), median (IQR)	240 (197–293)	241 (202–294)	211 (154–283)	0.024
Glucose (mg/dL), median (IQR)	131 (104–185)	131 (104–186)	137 (106–171)	0.928
Creatinine (mg/dL), median (IQR)	0.79 (0.64–0.99)	0.79 (0.64–0.96)	0.97 (0.69–1.44)	0.003
Urea (mg/dL), median (IQR)	41.0 (32.0–53.0)	40.0 (32.0–52.0)	57.0 (38.5–90.0)	<0.001
Albumin (g/L), median (IQR)	37.0 (34.0–40.0)	38.0 (34.0–40.0)	34.0 (29.5–37.5)	<0.001
Total bilirubin (mg/dL), median (IQR)	0.58 (0.42–0.79)	0.56 (0.41–0.77)	0.68 (0.48–0.95)	0.062
Total cholesterol (mg/dL), median (IQR)	179 (152–210)	179 (152–209)	174 (136–220)	0.652
Triglycerides (mg/dL), median (IQR)	129 (91–171)	130 (94–173)	110 (79–162)	0.098
HDL (mg/dL), median (IQR)	44.0 (38.0–52.0)	44.0 (38.0–52.0)	44.0 (35.5–54.5)	0.611
LDL (mg/dL), median (IQR)	105 (84–127)	105 (84–126)	107 (80–142)	0.748
CRP (mg/L), median (IQR)	5.37 (3.40–13.15)	5.20 (3.20–10.92)	18.50 (5.40–77.05)	<0.001
pH, median (IQR)	7.39 (7.35–7.42)	7.39 (7.35–7.42)	7.36 (7.31–7.42)	0.067
pO2 (mmHg), median (IQR)	37.5 (26.5–45.8)	37.3 (26.5–45.6)	38.5 (27.5–51.4)	0.340
pCO_2_ (mmHg), median (IQR)	43.9 (38.9–48.5)	44.0 (39.4–48.5)	41.7 (37.5–47.0)	0.107
HCO_3_ (mmol/L), median (IQR)	26.1 (23.8–28.5)	26.1 (24.0–28.5)	25.4 (21.4–28.3)	0.049
Lactate (mmol/L), median (IQR)	1.50 (1.10–1.90)	1.40 (1.10–1.80)	1.60 (1.30–2.50)	0.011

**Table 5:** Comparison of laboratory values in patients with ischemic stroke by mortality status.

Characteristic	Total (*n* = 389)	Survivors (*n* = 356)	Nonsurvivors (*n* = 33)	*p*
TyG index, mean ± SD	9.14 ± 0.69	9.15 ± 0.70	9.03 ± 0.62	0.350
PNI, mean ± SD	46.33 ± 7.18	46.82 ± 6.83	41.12 ± 8.77	0.001
WBC (×10^3^/mm^3^), median (IQR)	8.50 (7.00–10.60)	8.30 (6.90–10.30)	11.30 (8.70–13.15)	<0.001
Hemoglobin (g/dL), median (IQR)	13.90 (12.50–15.10)	13.90 (12.60–15.10)	12.70 (11.60–14.65)	0.071
Lymphocytes (×10^3^/mm^3^), median (IQR)	1.78 (1.29–2.35)	1.81 (1.32–2.37)	1.44 (0.97–2.15)	0.038
Neutrophils (×10^3^/mm^3^), median (IQR)	5.57 (4.30–7.74)	5.41 (4.26–7.36)	8.11 (5.85–11.46)	<0.001
Platelets (×10^3^/mm^3^), median (IQR)	240 (196–294)	240 (200–295)	211 (161–285)	0.073
Glucose (mg/dL), median (IQR)	131 (104–192)	130 (103–193)	139 (108–200)	0.707
Creatinine (mg/dL), median (IQR)	0.80 (0.66–1.00)	0.79 (0.64–0.97)	0.97 (0.74–1.44)	0.002
Urea (mg/dL), median (IQR)	41.0 (32.5–53.5)	40.0 (32.0–52.0)	61.0 (42.0–90.0)	<0.001
Albumin (g/L), median (IQR)	37.0 (34.0–40.0)	38.0 (34.0–40.0)	34.0 (28.5–37.0)	<0.001
Total bilirubin (mg/dL), median (IQR)	0.57 (0.42–0.79)	0.56 (0.41–0.78)	0.69 (0.51–0.95)	0.053
Total cholesterol (mg/dL), median (IQR)	177 (152–209)	179 (152–209)	169 (136–216)	0.458
Triglycerides (mg/dL), median (IQR)	130 (94–172)	132 (95–175)	115 (84–162)	0.242
HDL (mg/dL), median (IQR)	44.0 (37.0–52.0)	44.0 (38.0–52.0)	44.0 (35.5–52.5)	0.557
LDL (mg/dL), median (IQR)	105 (84–126)	105 (84–126)	106 (80–141)	0.986
CRP (mg/L), median (IQR)	5.31 (3.39–13.10)	5.20 (3.20–10.77)	30.00 (5.40–95.45)	<0.001
pH, median (IQR)	7.39 (7.35–7.42)	7.39 (7.35–7.42)	7.35 (7.31–7.42)	0.083
pO2 (mmHg), median (IQR)	36.9 (26.2–45.7)	36.8 (26.1–45.4)	38.5 (27.0–51.4)	0.321
pCO2 (mmHg), mean ± SD	44.0 ± 7.4	44.2 ± 7.1	42.3 ± 9.4	0.169
HCO3 (mmol/L), median (IQR)	26.0 (23.8–28.5)	26.1 (23.9–28.5)	25.3 (21.4–28.1)	0.039
Lactate (mmol/L), median (IQR)	1.40 (1.10–1.90)	1.40 (1.10–1.80)	1.60 (1.25–2.70)	0.027

**Table 6:** Comparison of laboratory values in patients with hemorrhagic stroke by mortality status.

Characteristic	Total (*n* = 26)	Survivors (*n* = 18)	Nonsurvivors (*n* = 8)	*p*
TyG index, mean ± SD	8.86 ± 0.68	8.94 ± 0.69	8.65 ± 0.63	0.321
PNI, mean ± SD	45.59 ± 7.48	48.17 ± 7.32	39.79 ± 3.79	0.006
WBC (×10^3^/mm^3^), median (IQR)	9.85 (8.43–14.18)	8.85 (8.15–13.60)	11.95 (9.72–16.12)	0.165
Hemoglobin (g/dL), mean ± SD	13.40 ± 2.11	13.46 ± 2.17	13.26 ± 2.13	0.835
Lymphocytes (×10^3^/mm^3^), median (IQR)	1.07 (0.89–2.35)	1.58 (0.91–2.40)	0.95 (0.53–1.10)	0.119
Neutrophils (×10^3^/mm^3^), median (IQR)	7.09 (5.14–12.50)	6.75 (4.71–10.16)	10.14 (7.43–14.36)	0.085
Platelets (×10^3^/mm^3^), mean ± SD	234 ± 77	253 ± 58	192 ± 100	0.142
Glucose (mg/dL), median (IQR)	136 (111–151)	136 (116–154)	134 (98–144)	0.291
Creatinine (mg/dL), median (IQR)	0.77 (0.62–0.96)	0.77 (0.62–0.90)	0.83 (0.56–2.00)	0.523
Urea (mg/dL), median (IQR)	40.5 (29.5–49.0)	37.0 (27.8–46.2)	46.0 (35.0–115.5)	0.173
Albumin (g/L), mean ± SD	37.3 ± 3.8	38.4 ± 3.2	34.8 ± 4.0	0.022
Total bilirubin (mg/dL), median (IQR)	0.61 (0.44–0.74)	0.61 (0.42–0.73)	0.55 (0.43–0.99)	0.802
Total cholesterol (mg/dL), mean ± SD	181 ± 47	183 ± 39	177 ± 64	0.771
Triglycerides (mg/dL), median (IQR)	98 (81–138)	106 (88–138)	91 (63–161)	0.374
HDL (mg/dL), mean ± SD	46.4 ± 12.5	47.7 ± 11.7	43.5 ± 14.7	0.439
LDL (mg/dL), mean ± SD	113 ± 44	113 ± 44	112 ± 46	0.971
CRP (mg/L), median (IQR)	7.55 (3.67–15.35)	7.04 (3.65–12.62)	9.87 (3.91–46.73)	0.222
pH, median (IQR)	7.40 (7.36–7.43)	7.41 (7.38–7.43)	7.38 (7.28–7.43)	0.253
pO_2_ (mmHg), median (IQR)	40.4 (33.1–57.6)	41.5 (37.4–57.6)	36.2 (28.7–89.2)	0.541
pCO_2_ (mmHg), mean ± SD	43.0 ± 6.0	43.3 ± 6.2	42.4 ± 5.9	
HCO_3_ (mmol/L), median (IQR)	27.6 (23.7–28.8)	27.6 (25.4–28.8)	27.0 (17.7–30.0)	0.697
Lactate (mmol/L), median (IQR)	1.55 (1.10–2.05)	1.30 (0.88–2.23)	1.75 (1.35–2.00)	0.343

**Table 7:** Comparison of laboratory values in the overall stroke cohort (ischemic and hemorrhagic stroke combined) by early mortality status.

Characteristic	Total (*n* = 406)	Survivors (*n* = 374)	Early mortality (*n* = 32)	*p*
TyG index, mean ± SD	9.13 ± 0.69	9.14 ± 0.70	9.03 ± 0.62	0.391
PNI, mean ± SD	46.49 ± 7.10	46.88 ± 6.85	41.86 ± 8.42	<0.001
WBC (×10^3^/mm^3^), median (IQR)	8.55 (7.10–10.60)	8.40 (7.00–10.32)	11.80 (9.20–15.78)	<0.001
Hemoglobin (g/dL), median (IQR)	13.90 (12.50–15.03)	13.90 (12.50–15.03)	12.95 (11.80–15.15)	0.418
Lymphocytes (×10^3^/mm^3^), median (IQR)	1.78 (1.26–2.35)	1.81 (1.30–2.36)	1.35 (0.95–2.15)	0.018
Neutrophils (×10^3^/mm^3^), median (IQR)	5.64 (4.37–7.76)	5.47 (4.28–7.40)	9.46 (6.29–13.59)	<0.001
Platelets (×10^3^/mm^3^), median (IQR)	240 (197–293)	241 (202–294)	207 (152–276)	0.013
Glucose (mg/dL), median (IQR)	132 (104–186)	131 (104–186)	139 (108–184)	0.709
Creatinine (mg/dL), median (IQR)	0.79 (0.64–0.99)	0.79 (0.64–0.96)	0.96 (0.72–1.46)	0.004
Urea (mg/dL), median (IQR)	41.0 (32.0–53.0)	40.0 (32.0–52.0)	60.5 (40.0–97.8)	<0.001
Albumin (g/L), median (IQR)	38.0 (34.0–40.0)	38.0 (34.0–40.0)	34.0 (30.0–37.8)	0.001
Total bilirubin (mg/dL), median (IQR)	0.58 (0.42–0.79)	0.56 (0.41–0.77)	0.69 (0.48–1.02)	0.058
Total cholesterol (mg/dL), median (IQR)	179 (152–210)	179 (152–209)	174 (142–227)	0.902
Triglycerides (mg/dL), median (IQR)	130 (92–171)	130 (94–173)	118 (81–166)	0.240
HDL (mg/dL), median (IQR)	44.0 (38.0–52.0)	44.0 (38.0–52.0)	44.0 (36.2–54.8)	0.813
LDL (mg/dL), median (IQR)	105 (84–127)	105 (84–126)	110 (84–146)	0.476
CRP (mg/L), median (IQR)	5.32 (3.39–13.03)	5.20 (3.20–10.92)	23.65 (5.39–101.80)	<0.001
pH, median (IQR)	7.39 (7.35–7.42)	7.39 (7.35–7.42)	7.36 (7.31–7.42)	0.021
pO_2_ (mmHg), median (IQR)	37.0 (26.5–45.7)	37.3 (26.5–45.6)	35.8 (26.6–49.1)	0.810
pCO_2_ (mmHg), median (IQR)	44.0 (39.2–48.5)	44.0 (39.4–48.5)	42.2 (38.1–48.3)	0.463
HCO_3_ (mmol/L), mean ± SD	26.1 ± 3.6	26.2 ± 3.6	24.8 ± 4.3	0.036
Lactate (mmol/L), median (IQR)	1.50 (1.10–1.90)	1.40 (1.10–1.80)	1.90 (1.40–2.68)	0.001

**Table 8:** Comparison of laboratory values in patients with ischemic stroke by early mortality status.

Characteristic	Total (*n* = 383)	Survivors (*n* = 356)	Early mortality (*n* = 27)	*p*
TyG index, mean ± SD	9.14 ± 0.69	9.15 ± 0.70	9.07 ± 0.60	0.586
PNI, mean ± SD	46.51 ± 7.07	46.82 ± 6.83	42.46 ± 8.90	0.002
WBC (x10^3^/mm3), median (IQR)	8.50 (7.00–10.50)	8.30 (6.90–10.30)	11.30 (9.00–15.10)	<0.001
Hemoglobin (g/dL), median (IQR)	13.90 (12.50–15.10)	13.90 (12.60–15.10)	12.80 (12.10–15.20)	0.362
Lymphocytes (×10^3^/mm^3^), median (IQR)	1.80 (1.29–2.36)	1.81 (1.32–2.37)	1.44 (0.95–2.15)	0.071
Neutrophils (×10^3^/mm^3^), median (IQR)	5.53 (4.29–7.71)	5.41 (4.26–7.36)	9.38 (6.18–12.07)	<0.001
Platelets (×10^3^/mm^3^), median (IQR)	240 (197–293)	240 (200–295)	211 (156–276)	0.030
Glucose (mg/dL), median (IQR)	131 (104–193)	130 (103–193)	149 (110–212)	0.394
Creatinine (mg/dL), median (IQR)	0.79 (0.65–0.99)	0.79 (0.64–0.97)	0.92 (0.77–1.41)	0.006
Urea (mg/dL), median (IQR)	41.0 (32.0–53.0)	40.0 (32.0–52.0)	64.0 (39.0–91.0)	<0.001
Albumin (g/L), median (IQR)	38.0 (34.0–40.0)	38.0 (34.0–40.0)	34.0 (30.0–38.0)	0.005
Total bilirubin (mg/dL), median (IQR)	0.57 (0.41–0.79)	0.56 (0.41–0.78)	0.69 (0.54–1.00)	0.064
Total cholesterol (mg/dL), median (IQR)	178 (152–210)	179 (152–209)	174 (146–222)	0.992
Triglycerides (mg/dL), median (IQR)	130 (92–172)	132 (95–175)	115 (79–155)	0.213
HDL (mg/dL), median (IQR)	44.0 (37.0–52.0)	44.0 (38.0–52.0)	44.0 (37.0–55.0)	0.819
LDL (mg/dL), median (IQR)	105 (85–126)	105 (84–126)	108 (85–145)	0.518
CRP (mg/L), median (IQR)	5.27 (3.39–12.70)	5.20 (3.20–10.77)	18.50 (5.38–111.00)	<0.001
pH, median (IQR)	7.39 (7.35–7.42)	7.39 (7.35–7.42)	7.35 (7.32–7.42)	0.070
pO_2_ (mmHg), median (IQR)	36.8 (26.1–45.4)	36.8 (26.1–45.4)	35.6 (26.3–49.3)	0.791
pCO_2_ (mmHg), median (IQR)	44.0 (39.2–48.7)	44.0 (39.4–48.7)	42.0 (38.0–48.5)	0.497
HCO_3_ (mmol/L), mean ± SD	26.1 ± 3.6	26.1 ± 3.6	24.9 ± 3.7	0.094
Lactate (mmol/L), median (IQR)	1.50 (1.10–1.90)	1.40 (1.10–1.80)	1.90 (1.40–2.70)	0.002

**Table 9:** Comparison of laboratory values in patients with hemorrhagic stroke by early mortality status.

Characteristic	Total (*n* = 23)	Survivors (*n* = 18)	Early Mortality (*n* = 5)	*p*
TyG index, mean ± SD	8.91 ± 0.69	8.94 ± 0.69	8.79 ± 0.74	0.664
PNI, mean ± SD	46.09 ± 7.82	48.17 ± 7.32	38.60 ± 4.35	0.012
WBC (×10^3^/mm^3^), median (IQR)	9.40 (8.20–13.90)	8.85 (8.15–13.60)	13.60 (8.55–16.65)	0.290
Hemoglobin (g/dL), mean ± SD	13.52 ± 2.21	13.46 ± 2.17	13.76 ± 2.61	0.792
Lymphocytes (×10^3^/mm^3^), median (IQR)	1.28 (0.92–2.35)	1.58 (0.91–2.40)	0.95 (0.55–1.87)	0.446
Neutrophils (×10^3^/mm^3^), median (IQR)	6.96 (5.10–12.13)	6.75 (4.71–10.16)	11.51 (6.16–15.45)	0.174

**Table 10:** Performance of PNI for predicting in-hospital mortality.

Index	AUC (95% CI)	Cutoff	Sensitivity (%)	Specificity (%)	Positive Predictive Value (%)	Negative Predictive Value (%)	*p*
PNI	In-hospital mortality in the overall stroke cohort (ischemic and hemorrhagic stroke)						
	0.738 (0.652–0.824)	45.3	78	60.2	17.7	96.2	<0.001
	In-hospital mortality in patients with ischemic stroke						
	0.716 (0.613–0.819)	40.5	54.5	82.3	22.2	95.1	<0.001
	In-hospital mortality in patients with hemorrhagic stroke						
	0.872 (0.735–1)	43.75	100	72.2	61.5	100	<0.001

**Table 11:** Univariable and multivariable logistic regression analysis of factors associated with in-hospital mortality.

Variable	Univariable OR	95% CI	*p*	Multivariable OR	95% CI	*p*
**PNI (per 1 unit)**	0.876	0.831–0.923	**<0.001**	0.933	0.877–0.993	**0.029**
**Age (years)**	1.055	1.021–1.089	**0.001**	1.054	1.014–1.095	**0.008**
**Stroke type**						
Ischemic	Reference	–		Reference	–	
Hemorrhagic vs. ischemic	4.768	1.927–11.797	**<0.001**	7.154	2.594–19.729	**<0.001**
**CRP (per 1 mg/L)**	1.013	1.007–1.019	**<0.001**	1.010	1.004–1.016	**0.002**

Multivariable analysis was performed with the backward method including PNI, age, stroke type, and CRP (*n* = 413; 41 in-hospital deaths; two patients with missing CRP were excluded). Model fit: omnibus χ^2^ = 52.4, df = 4, *p* < 0.001; Hosmer–Lemeshow *p* = 0.422; Nagelkerke R^2^ = 0.250; AUC = 0.777. OR, odds ratio; CI, confidence interval; PNI, prognostic nutritional index; CRP, C-reactive protein.

## Data Availability

The data may be available from the corresponding author upon reasonable request and subject to institutional approval.
